# The complete mitochondrial genome of *Oestrus ovis* (Linnaeus, 1758) (Diptera: Oestridae)

**DOI:** 10.1080/23802359.2021.1934174

**Published:** 2021-06-03

**Authors:** Gaël Aleix-Mata, Jesús M. Peréz, Antonio Sánchez

**Affiliations:** a Department of Experimental Biology, Jaén University, Jaén, Spain; b Department of Animal and Plant Biology, and Ecology, Jaén University, Jaén, Spain; c Wildlife Ecology & Health Group (WE&H), Jaén, Spain

**Keywords:** Complete mitochondrial genome, *Oestrus ovis*, Oestridae, Diptera

## Abstract

Larvae of the sheep bot fly, *Oestrus ovis* (Linnaeus, 1758), cause cavitary myiasis in domestic and wild hosts, including man. The complete circular *O. ovis* mitogenome was assembled, which is 16,584 bp in length and encodes 13 protein-coding genes, 22 tRNA genes, and two rRNA genes. The phylogenetic tree of *O. ovis* and 13 related Oestridae species, with *Sarcophaga tuberosa* as outgroup, was built.

The family Oestridae includes 29 genera grouped into four subfamilies with about 170 species (Scholl et al. 2019). Oestrids are obligate parasites in their larval phases, their infestations can increase the likelihood of opportunistic infections and in severe cases can lead to head injury and even death of the host (Mozaffari et al. 2013). In this study, we sequenced and analyzed for the first time the complete mitochondrial genome of *Oestrus ovis.*

The larvae of *O. ovis* were obtained during the necropsy of a domestic goat (*Capra hircus*) (Almería, Spain: 37°03′05.5″N − 2°34′06.1″W). The larvae were identified based on morphological criteria, particularly the posterior peritremes and dorsal spinulation (Zumpt 1965; Wetzel and Bauristhene 1970). Total DNA was extracted from one larva with the Quick-DNA Tissue/Insect kit (Zymo Research, Irvine, CA) and 20 Gbp of sequences were obtained using the Illumina^®^ Hiseq™ 2000 platform in paired-end reads with length 2 × 100 nt. A specimen was deposited in the Department of Experimental Biology at the Jaén University (https://www.ujaen.es/departamentos/bioexp; abaca@ujaen.es) under the reference Ovinoca1.

Previous to the assembly, we removed sequencing adaptors and low quality reads (threshold of Q20) to get read pairs meeting the quality criteria in all their nucleotides using the Trimmomatic program (Bolger et al. 2014). Next, we randomly selected a million read pairs using seqTK (https://github.com/lh3/seqtk). The mitogenome was assembled with MITObim v1.8 program (Hahn et al. 2013) using the selected reads and the mitogenome of *Rhinoestrus usbekistanicus* (accession number MN833259; Li et al. 2020) as a reference. For this step, we applied the ‘–quick’ option and 15% as mismatch threshold. The *O. ovis* mitogenome was annotated with MITOS (Bernt et al. 2013) and tRNA scan-SE (Lowe and Eddy 1997). Finally, the annotations of the genes were refined by manual comparison with the mitogenomes of close species.

The length of the mitochondrial genome is 16,584 bp, with an overall of 75.4% AT content (GenBank accession number MW145179). The nucleotide distribution for the mitochondrial genome is 39.6% A, 15.9% C, 8.3% G, and 36.2% T. It contains typically 13 protein-coding genes (PCGs), 22 transfer RNAs, two ribosomal RNAs, and one control region (D-loop). Twenty-three of the 37 genes were encoded on the heavy strand while the 14 were on the light strand. Regarding the PCGs, nine (ATP6, ATP8, COB, COX1, COX2, COX3, NAD2, NAD3, and NAD6) are transcribed on the heavy strand and the remaining four (NAD1, NAD4, NAD4L, and NAd5) on the light strand. The two rRNA genes are transcribed on the light strand, the 22 transfer RNAs, are found discontinuously scattered in the entire mitochondrial genome and 14 are in the heavy strand and eight in the light strand.

In addition, we analyzed the phylogenetic relationships of *O. ovis*. For this analysis, the whole mitochondrial genome of the 13 Oestridae species currently available in GenBank were used, and the genome of *Sarcophaga tuberosa* (Zhang et al. 2019; MK820723) as outgroup. For the phylogenetic analysis, sequence alignment was performed with Clustal Omega (Sievers et al. 2011), the poorly aligned positions and divergent regions were removed using Gblocks program v0.91b (Talavera and Castresana 2007). The phylogenetic tree was built by the maximum-likelihood method with 1000 replicates using MEGAX software (Kumar et al. 2018). The results showed conventional taxon pattern, previously established for the family Oestridae, and shows *O. ovis* as sister to *R. usbekistanicus* (Figure 1).

**Figure 1.**
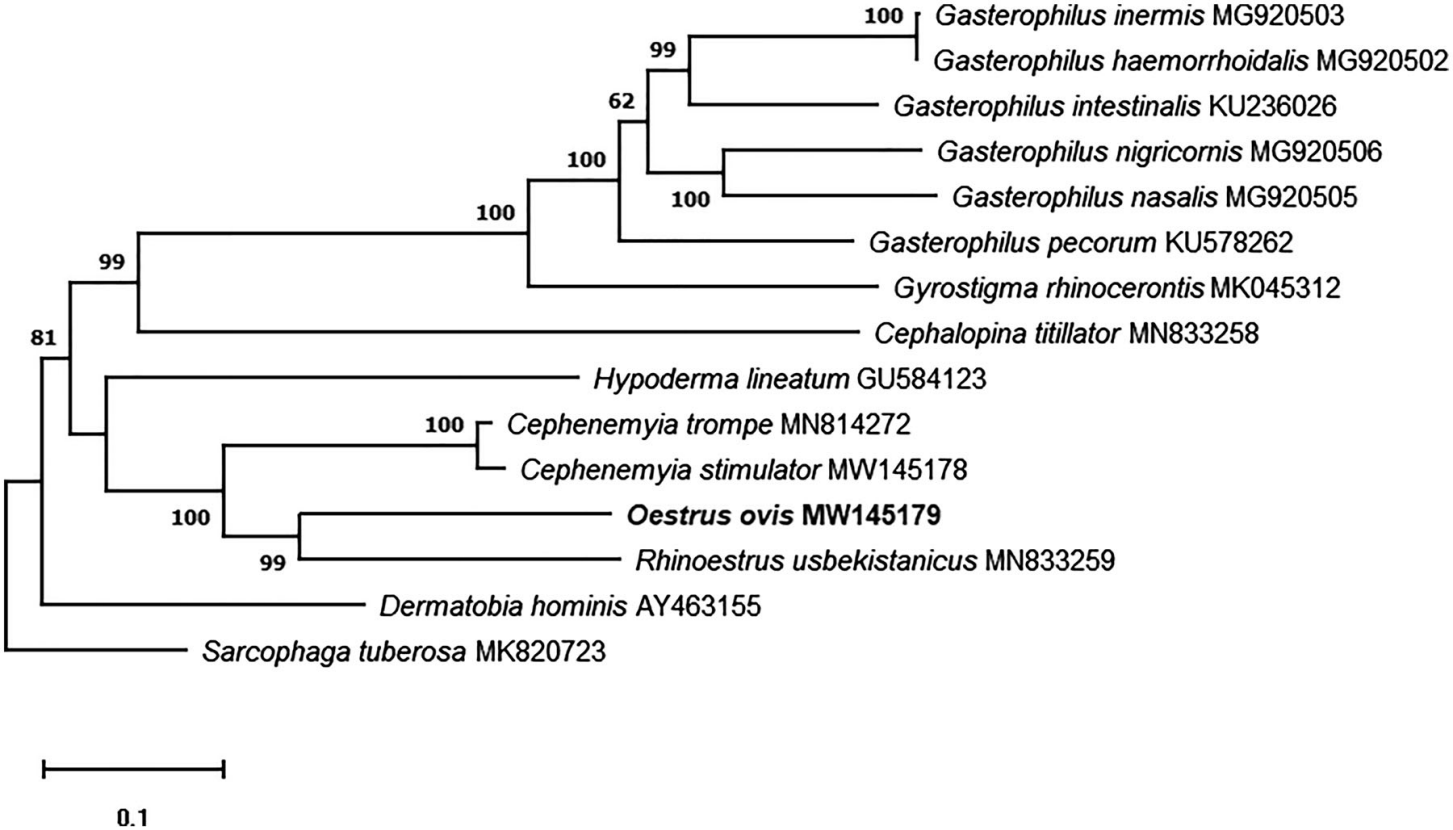
Maximum-likelihood phylogenetic tree based on the complete mitochondrial genomes of 13 Oestridae species and *Sarcophaga tuberosa* as outgroup. Nodal numbers represent bootstrap support with 1000 bootstrap replicates.

## Data Availability

The data that support the findings of this study are openly available in the US National Center for Biotechnology Information (NCBI database) at https://www.ncbi.nlm.nih.gov/, reference number: MW145179.
